# Robust AOA-Based Target Localization for Uniformly Distributed Noise via *ℓ_p_*-*ℓ*_1_ Optimization

**DOI:** 10.3390/e24091259

**Published:** 2022-09-07

**Authors:** Yanping Chen, Chunmei Wang, Qingli Yan

**Affiliations:** 1 School of Computer Science and Technology, Xi’an University of Posts and Telecommunications, Xi’an 710121, China; 2 Shaanxi Key Laboratory of Network Data Analysis and Intelligent Processing, Xi’an University of Posts and Telecommunications, Xi’an 710121, China; 3 Xi’an Key Laboratory of Big Data and Intelligent Computing, Xi’an 710121, China

**Keywords:** alternating direction method of multipliers, angle of arrival, *ℓp*-norm, outliers, sparse regularization

## Abstract

This paper addresses the problem of robust angle of arrival (AOA) target localization in the presence of uniformly distributed noise which is modeled as the mixture of Laplacian distribution and uniform distribution. Motivated by the distribution of noise, we develop a localization model by using the $\ell_{p}$-norm with 0≤p<2 as the measurement error and the $\ell_{1}$-norm as the regularization term. Then, an estimator for introducing the proximal operator into the framework of the alternating direction method of multipliers (POADMM) is derived to solve the convex optimization problem when 1≤p<2. However, when 0≤p<1, the corresponding optimization problem is nonconvex and nonsmoothed. To derive a convergent method for this nonconvex and nonsmooth target localization problem, we propose a smoothed POADMM estimator (SPOADMM) by introducing the smoothing strategy into the optimization model. Eventually, the proposed algorithms are compared with some state-of-the-art robust algorithms via numerical simulations, and their effectiveness in uniformly distributed noise is discussed from the perspective of root-mean-squared error (RMSE). The experimental results verify that the proposed method has more robustness against outliers and is less sensitive to the selected parameters, especially the variance of the measurement noise.

## 1. Introduction

With the rapid development of wireless sensor networks, wireless sensor network localization technology, as one of its key technologies, has also been widely studied and applied [1,2,3,4,5,6,7,8,9,10,11]. The typical target localization techniques for wireless sensor networks use multiple sensor nodes to receive target radiation signals and obtain accurate localization parameter information from them, then transmit them to the processing center for fusion processing and achieve the solution of the target position by establishing a series of localization equations. According to the different positioning parameter information obtained by the sensors, the target localization technique is roughly divided into received signal strength (RSS)-based localization [4,5], time of arrival (TOA)-based localization [6,7], time difference of arrival (TDOA)-based localization [8,9], and angle of arrival (AOA)-based localization [10,11].

The RSS-based localization technique measures the average power of the received signal and then locates the target based on a known wireless channel attenuation model. The channel attenuation model has a significant impact on the positioning accuracy of RSS, which is often poor because the actual signal propagation environment is very complex and often time-varying, making it difficult to establish a signal attenuation model that matches the actual situation. The TOA-based localization technique uses the transmission time delay of the target signal reaching each sensor node times the signal propagation speed to estimate the distance between the target and the sensor. The distance is used to determine a series of circles centered on the node positions, and the intersection of the circles is the target position. The TDOA-based localization technique measures the time difference between the target signal reaching two sensor nodes. Using the time difference, a series of hyperbolas with the sensor position as the focal point is determined, and the intersection point of these hyperbolas is the position of the target. However, both TOA and TDOA need to maintain clock synchronization between sensors or between sensors and targets. The AOA-based localization technique estimates the AOA of the target by each sensor node, and then can obtain a straight line with AOA as the slope from the node position, and the intersection of all the lines is the target position. Each sensor node is equipped with an antenna array to estimate the angular values, making the AOA localization technique more demanding on the hardware of the nodes. However, with the advent of low-cost, low-power, high-performance directional antennas in recent years, their cost is rapidly decreasing and this is no longer a limiting factor for the application of AOA localization techniques. Since each node performs AOA estimation individually, the signals at different nodes do not need to be synchronized. Moreover, the algorithm has simple computational principles and methods, low communication overhead, and high localization accuracy. Therefore, the AOA-based localization technique has received extensive attention and research.

Target localization using the angle of arrival (AOA) has attracted considerable interest for several decades due to its wide application in civil and military fields, such as detection and surveillance, radar, and sonar [12,13,14,15,16]. The AOA-based localization method does not require strict clock synchronization of individual nodes and has high localization accuracy and simple computational principles. We can estimate the target position by collecting bearing measurements from the transmitted signals of multiple spatially distributed sensors.

The AOA-based positioning technology has rapidly grown into a research hotspot and proposed many effective methods, such as maximum likelihood estimator (MLE), pseudolinear estimator (PLE), and total least squares (TLS) estimator [17,18,19]. Among these methods, the MLE and PLE methods are the most commonly used. MLE is an asymptotically unbiased and efficient estimator, it does not have a closed-form solution and has to be implemented in an iterative search way [17]. PLE which does not require a Gaussian noise assumption is easy to implement with low complexity [18]. It is worth noting that these methods perform well under the assumption that the bearing measurement error follows a Gaussian distribution. However, the practical application environment is complex, and the measurement noise may have different kinds of combinations that exhibit non-Gaussian characteristics [20,21]. In addition, sensor nodes are most deployed in harsh environments, vulnerable to impulsive noise and non-line of sight (NLOS) propagation, external attacks and natural causes, or physical failures [22,23,24,25]. All these interferences and limitations may result in unreliable measurements, also called outliers, which can significantly degrade the localization performance.

There are two traditional methods to handle outlier problems: outlier detection and robust estimator. Outlier detection refers to detecting suspected outliers and separating them from the original data set, then using the remaining data to complete the localization [26,27,28]. Although the outlier detection method is intuitive and effective, it is not suitable for large data sets or complex application scenarios. The robust estimator approach is different from the outlier detection approach, which incorporates all the measurements into an estimator against outliers, enabling robust location estimation without pre-processing the data [29,30]. Recently, motivated by the sparse property of outliers, the localization method based on sparse regularization is proposed to simultaneously estimate the target position and outliers [31,32]. The main attraction of the sparse promoting method is that it can estimate source position and outliers jointly, and in the process of alternating updating, the source location is estimated by the modified outlier measurements instead of discarding them. The proposed methods use the $\ell_{2}$-norm as the measurement error, which is based on the mixture distribution of Gaussian and uniform. In this paper, we model the noise distribution as the mixture of Laplacian and uniform distribution. Under this noise assumption, the $\ell_{2}$-loss function would not be robust for the Laplacian component.

To overcome this problem, we propose a robust localization method based on the $\ell_{p}$-$\ell_{1}$$\left(0\leq p<2\right)$ optimization in this letter. Compared with the $\ell_{2}$-norm, the $\ell_{p}$-norm is less sensitive to large outliers when p<2, especially when *p* is small. The main contributions of this paper can be summarized as follows:

1. The sparse regularized robust model based on the $\ell_{p}$-$\ell_{1}$$\left(0\leq p<2\right)$ optimization is proposed for the target localization in the presence of uniformly distributed noise, which enables the joint estimation of outliers and target position;
2. The optimization algorithm for introducing the proximal operator into the framework of alternating direction method of multipliers (POADMM) is developed to solve the model effectively when 1≤p<2;
3. To derive a convergent algorithm, a smoothed POADMM (SPOADMM) optimization algorithm is presented by a smoothing strategy for the nonconvex case of 0≤p<1.

The rest of this paper is organized as follows. The related work on outlier-oriented AOA-based techniques is reviewed in Section 2. We first introduce the AOA measurement model and the localization model associated noise in Section 3. Two effective optimization algorithms, POADMM and SPOADMM, are derived in Section 4. Next, Section 5 conducts numerical experiments. Finally, Section 6 ends the paper.

## 2. Related Work

In this section, related work on outlier-oriented AOA-based techniques is briefly reviewed. In practical applications, the presence of outliers is inevitable, and the presence of outliers can significantly degrade the performance of positioning methods. Therefore, how to maintain the localization performance in the presence of outliers has attracted a lot of scholars’ research in recent years.

One of the typical methods is outlier detection, which eliminates suspicious outlier data in the measured data. A weighted linear least squares estimator is derived for the line of sight (LOS) AOA component in terms of the direction-of-arrival vector, and then a data selection method is performed using the sum of squared residuals to discard error-prone NLOS connections [26]. A Bayesian sequence test is first used to distinguish between the line of sight ( LOS ) or NLOS conditions. Based on the identified measurement condition, a modified Kalman filter (MKF) is used to smooth the measurement range and mitigate the NLOS effect. The distance estimated by MKF uses the residual weighting algorithm to calculate the target position [27]. A method for identifying unreliable orientation measurements is proposed. The target position is calculated by detecting outliers from a set of estimated positions obtained from different sensor combinations, filtering them out, and thus using the estimated positions obtained from reliable sensors [28]. An EM-based method is introduced into the AOA-based positioning method to achieve accurate positioning results by identifying unreliable measurement results from the NLOS propagation environment and discarding them [33].

Another typical method is the robust estimator. The well-known robust estimator is the M-estimator, which has been widely used to deal with outlier problems. An M-estimator based on Tukey and Huber functions is presented to solve the problem of AOA localization with unreliable measurements [29]. The algorithm can reject outliers when choosing an appropriate threshold. A robust structural global least squares algorithm for passive localization is proposed, which uses an improved Danish weight function to suppress the effect of outliers on the localization performance [30]. A distributed robust localization algorithm based on sensor network energy information is proposed. The algorithm uses the Bi-square function as the cost function of M-estimate [34].

The reliability of node observations and the estimated target location are mutually influenced. Accurate target position estimation depends on accurate reliability identification, which in turn affects reliability judgment. Therefore, it is an effective means to improve the positioning performance by proposing a synchronization estimation method that can realize the reliable evaluation of node observations and target location. The clustering information of intersections is introduced into the outlier tracking regularization method to estimate outlier and source locations simultaneously [31]. A robust AOA target localization method based on generalized minimal concave penalty (GMC) in the presence of correlated noise and outliers is proposed, which can estimate both outliers and target locations [32]. However, those methods all use the $\ell_{2}$-norm as the measurement error, which is well-known to be highly sensitive to outliers in the bearing observations. In this paper, we propose a robust localization method based on the $\ell_{p}$-$\ell_{1}$ (0≤p<2) optimization in uniformly distributed noise. Among them, the $\ell_{1}$-norm penalty is more tractable due to its convexity and hence most widely used in sparse optimization. Meanwhile, the $\ell_{p}$-norm is less sensitive to large outliers compared with the $\ell_{2}$-norm.

## 3. AOA-Based Localization Methods and Problem Statement

This section introduces the localization principle based on AOA measurement, which allows estimating the target location from the angles measured by multiple sensors. In addition, since the reliability of node observations and the estimated target location are mutually influenced, we propose a simultaneous estimation model that enables outlier identification and target position localization.

### 3.1. Angle of Arrival Model

**Figure 1:**
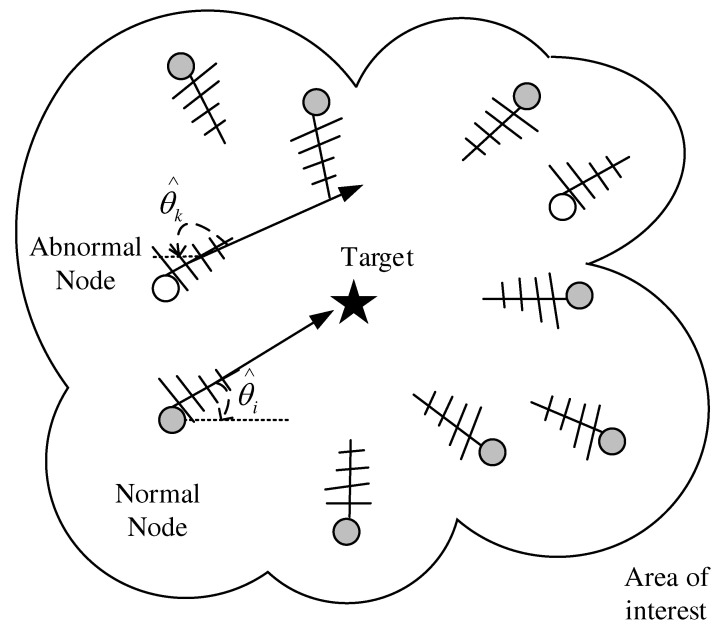
2-D AOA target localization geometry.

**Figure 2:**
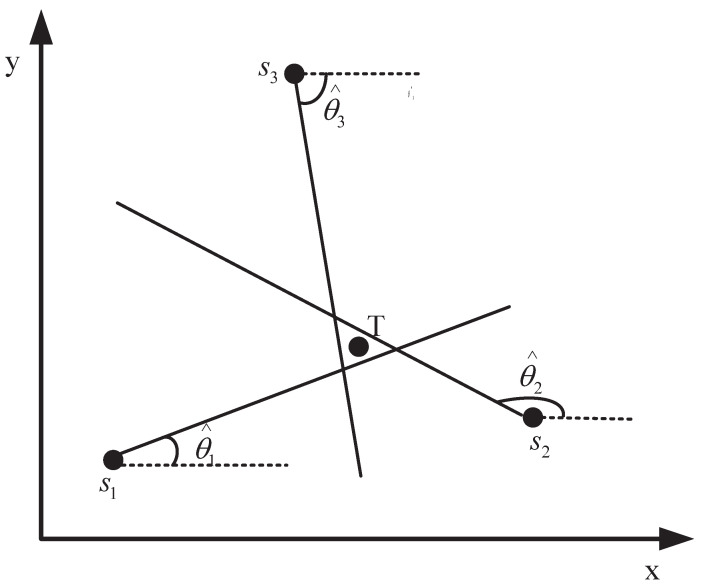
Illustration of an AOA localization system in 2-D space.

In space, the sensor nodes and targets are located as shown in 2-D space in Figure 1, and the AOA of the signals is collected by the antenna arrays equipped with the sensors. It is needed to emphasize that normal sensors acquire correct measurements, while abnormal sensors acquire wrong observations due to object occlusion, interference or network attacks, etc. Abstracting these to the plane, the AOA target localization problem is shown in Figure 2. Let $T={\left[x,y\right]}^{T}$ denotes the estimated unknown target location and multiple sensors locate at $s_{i}={\left[x_{i},y_{i}\right]}^{T},i=1,2,\cdots ,N$, *N* denotes the number of nodes in the sensor network.

Ideally, the true bearing $\theta_{i}$ can be expressed as

(1) $$\theta_{i}=\tan^{-1}\left(\frac{y-y_{i}}{x-x_{i}}\right),i=1,2,\cdots ,N,$$

where $\tan^{-1}$ stands for the four-quadrant arctangent and $\theta_{i}\in \left(0,2\pi \right]$.

In fact, the observations are not necessarily equal to $\theta_{i}$ and are usually affected by the measurement noise [35]

(2) $${\hat{\theta }}_{i}=\theta_{i}+\varepsilon_{i},i=1,2,\cdots ,N,$$

where $\varepsilon_{i}$ represents the node measurement error.

**Figure 3:**
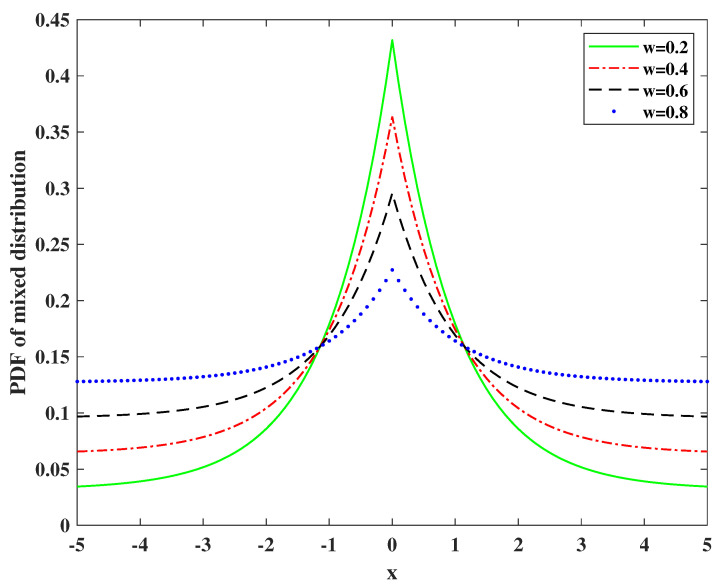
The pdf of the mixed distribution.

Considering that outliers caused by NLOS, impulsive noise, etc., may be any values within the range of $\left[-\pi ,\pi \right)$, we assume that the estimated bearings are contaminated by a uniform distribution with probability. In this paper, we use the mixture of Laplacian and uniform distribution as the measurement noise distribution [36,37,38].

(3) $$F=\left(1-w\right)La\left(\theta_{i},\sigma_{i}^{2}\right)+wU\left(-\pi ,\pi \right),$$

where *w* is the outlier occurrence probability, $La\left(\theta_{i},\sigma_{i}^{2}\right)$ represents the probability density function (pdf) of Laplacian distribution and the formula is given by $La\left(x|\theta ,\sigma^{2}\right)=\frac{1}{2\sigma^{2}}\exp\left(-\frac{\left|x-\theta \right|}{\sigma^{2}}\right)$, and U(−π,π) represents the pdf of uniform distribution. Figure 3 shows the pdf of the mixed distribution. The uniform distribution over the allowable range describes that we are blind to the possible measurement value, and the Laplacian component gives a sharp peak around the true value due to the deviations caused by normal background noise. The mixed model has been proved to belong to the heavy-tailed distribution class [28].

The measurement set from *N* nodes can be written in vector form as follows.

(4) $$\hat{\theta }=\theta +\varepsilon ,$$

where $\hat{\theta }={\left[{\hat{\theta }}_{1},{\hat{\theta }}_{2},\cdots ,{\hat{\theta }}_{N}\right]}^{T}$, $\theta ={\left[\theta_{1},\theta_{2},\cdots ,\theta_{N}\right]}^{T}$, $\varepsilon ={\left[\varepsilon_{1},\varepsilon_{2},\cdots ,\varepsilon_{N}\right]}^{T}$.

The nonlinear angle measurement (4) can be rewritten in pseudolinear form as [32]

(5) y=Ax+n,

where $A=\left[\begin{array}{cc} \sin\hat{\theta } & -\cos\hat{\theta } \end{array}\right]$, $y=[x\sin\hat{\theta }$-$y\cos\hat{\theta }]$, $n={\left[r_{1}\sin\varepsilon_{1},r_{2}\sin\varepsilon_{2},\ldots ,r_{N}\sin\varepsilon_{N}\right]}^{T}$$\approx {\left[r_{1}\varepsilon_{1},r_{2}\varepsilon_{2},\ldots ,r_{N}\varepsilon_{N}\right]}^{T}$ is the residual of pseudolinear Equation (5).

Then, the target position can be estimated by the PLE method, which is also known as an orthogonal vector estimator. The PLE requires that **x** is estimated by minimizing $\frac{1}{2}{∥y-\mathrm{Ax}∥}_{2}^{2}$ in the sense of $\ell_{2}$-norm optimization, the solution is

(6) $${\hat{x}}_{\mathrm{PLE}}={\left(A^{T}A\right)}^{-1}A^{T}y.$$

It is easy to implement with low complexity and is the most widely used model for Gaussian noise.

### 3.2. Problem Statement

This paper focuses on the problem of robust AOA localization in 2-D space in the presence of uniformly distributed measurement noise. Under such a scenario, the performance of the aforementioned PLE may be severely degraded since the $\ell_{2}$-norm optimization is sensitive to outliers.

We consider o to be the outlier vector, and the pseudolinear equation can be remodeled as

(7) y=Ax+n+o,

where $o={\left[o_{1},o_{2},\cdots ,o_{N}\right]}^{T}$ models the outliers, and $o_{i}$ is non-zero represents ${\hat{\theta }}_{i}$ is an outlier.

To estimate o and x simultaneously, we consider standardizing the linear model by exploiting the sparsity of the outliers. The typical approach to eliminate impulsive noise is to use the $\ell_{1}$-norm for both the residual and the regularization term. However, the $\ell_{1}$-norm tends to underestimate high-amplitude components of $x\in R^{N}$, which is one of its drawbacks [39]. As an alternative to $\ell_{1}$-norm, we use the $\ell_{p}$-norm $\left(0\leq p<2\right)$ as the residual term and propose the following formulation

(8) $$\left[\hat{x},\hat{o}\right]=\arg\min_{x,o}\frac{1}{2}{∥y-Ax-o∥}_{p}^{p}+\lambda {∥o∥}_{1},$$

where λ>0 is a regularization parameter to balance the residual and regularization terms.

**Figure 4:**
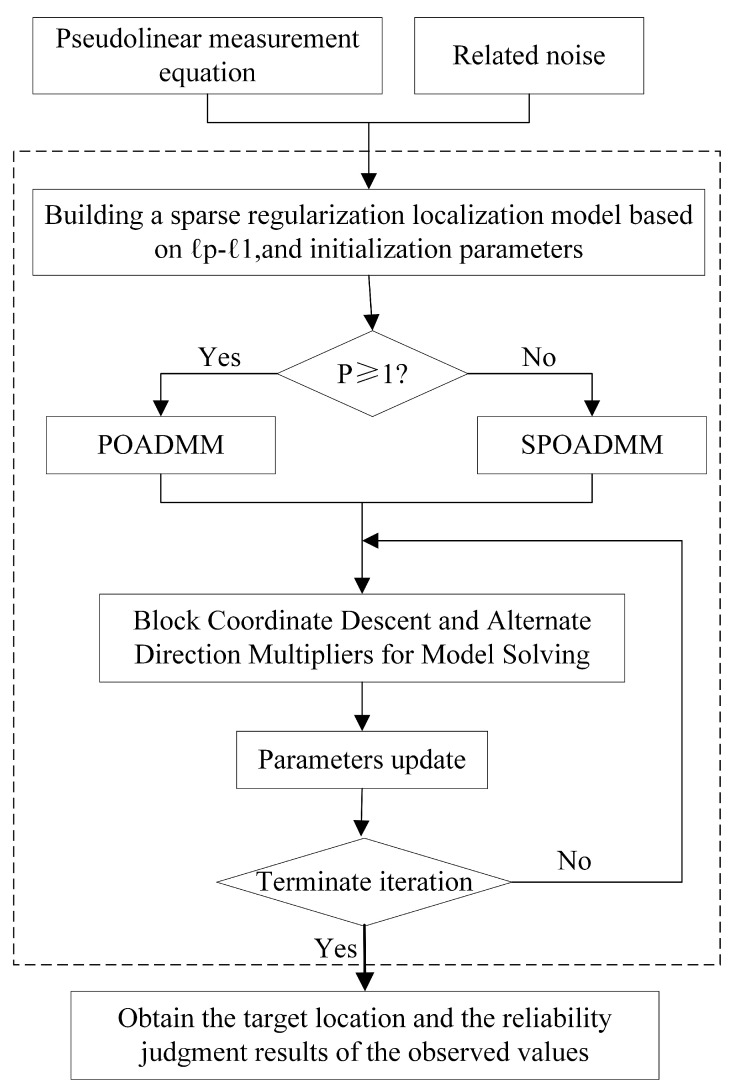
Illustration of the system framework.

When 1≤p<2, (8) is a convex optimization problem. For 0≤p<1, the objective in (8) is nonconvex and nonsmooth. Unlike convex optimization problems where convergence has been shown, non-convex optimization problems are much more challenging. We will develop corresponding estimators for these two optimization problems. Figure 4 depicts the system framework of this paper. 

## 4. Proposed Estimators

To solve the optimization problem (8), we can use proximal splitting-based algorithms, such as the alternating direction method of multipliers (ADMM) [40] or the forward-backward splitting (FBS) [41]. Considering that the optimization problem (8) is under-determined, which gives rise to motivation to estimate source position and bias jointly by ADMM.

### 4.1. POADMM Estimator

For Formula (8), the $\ell_{p}$-norm loss term and the $\ell_{1}$-norm regularization term are naturally separated using ADMM, which decouples the variables and makes the problem easy to solve. By introducing an auxiliary variable v, (8) can be equivalently reformulated as

(9) $$\begin{array}{c} \arg\min_{x,o}\frac{1}{2}{∥v∥}_{p}^{p}+\lambda {∥o∥}_{1}\\ \;s.t.y-Ax-o=v. \end{array}$$

We define the augmented Lagrangian function of (9),

(10) $$\begin{array}{c} L\left(v,o,\tau ,x\right)=\frac{1}{2}{∥v∥}_{p}^{p}+{\lambda ∥o∥}_{1}-<\tau ,y-Ax-o-v>+\frac{\rho }{2}{∥y-Ax-o-v∥}_{2}^{2} \end{array},$$

where τ is the Lagrange multiplier associated with the constraint in (9), ρ>0 is the penalty parameter. It is clearly that the ADMM solves (10) by alternating minimization with respect to the subproblems (v, o, x) and updating the dual variable τ.

(11) $$v^{k+1}=\arg\min_{v}L\left(v,o^{k},\tau^{k},x^{k}\right),$$

(12) $$o^{k+1}=\arg\min_{o}L\left(v^{k+1},o,\tau^{k},x^{k}\right),$$

(13) $$\tau {}^{k+1}=\tau^{k}-\rho \left(y-Ax^{k}-o^{k+1}-v^{k+1}\right),$$

(14) $$x^{k+1}=\arg\min_{x}L\left(v^{k+1},o^{k+1},\tau^{k+1},x\right).$$

Then, we will discuss the minimization of each subproblem. First, the v-subproblem (11) can be written as

(15) $$v^{k+1}=\arg\min_{v}\left(\frac{1}{2}{∥v∥}_{p}^{p}+\frac{\rho }{2}{∥y-Ax^{k}-o^{k}-v-\frac{\tau^{k}}{\rho }∥}_{2}^{2}\right).$$

As can be noted, (15) can be regarded as the $\ell_{p}$$\left(1\leq p<2\right)$ regularization problem. When 1<p<2, the proximity operator of $\ell_{p}$-norm [42] can be used to solve as

(16) $$v^{k+1}=\mathrm{sign}\left(b^{k}\right)r^{k},$$

where $b^{k}=y-Ax^{k}-o^{k}-\frac{\tau^{k}}{\rho }$, $r^{k}$ is the solution of

(17) $$h_{1}\left(r\right)=\frac{1}{2}{pr}^{p-1}+\rho r-\rho \left|b^{k}\right|=0,r\geq 0.$$

It can be seen that $h_{1}\left(r\right)$ is an increasing and concave function for r≥0. Due to $h_{1}\left(0\right)<0$ and $h_{1}\left(b^{k}\right)>0$ when $b^{k}\neq 0$, so when $b^{k}\neq 0$, $r^{k}$ satisfies $0<r^{k}<\left|b^{k}\right|$ and can be computed by Newton’s method. When p=1 [43], $v^{k+1}$ can be solved by

(18) $$v^{k+1}=\mathrm{sign}\left(b^{k}\right)\max\left\{\left|b^{k}\right|-\frac{1}{2\rho },0\right\}.$$

The o-subproblem (12) can be viewed as

(19) $$o^{k+1}=\arg\min_{o}\lambda {∥o∥}_{1}+\frac{\rho }{2}{∥y-Ax^{k}-o-v^{k+1}-\frac{\tau^{k}}{\rho }∥}_{2}^{2}.$$

(19) is a $\ell_{2}$-$\ell_{1}$ minimization problem, which can obtain a closed-form solution by thresholding function [44]

(20) $$o^{k+1}=\mathrm{sign}\left(c^{k}\right)\max\left(\left|c^{k}\right|-\frac{\lambda }{2\rho },0\right),$$

where $c^{k}=y-Ax^{k}-v^{k+1}-\frac{\tau^{k}}{\rho }$. The x-subproblem (14) can be solved as

(21) $$x^{k+1}=\arg\min_{x}\frac{\rho }{2}{∥y-Ax-o^{k+1}-v^{k+1}-\frac{\tau {}^{k+1}}{\rho }∥}_{2}^{2}.$$

Solving for x amounts to a least squares problem. We have

(22) $$x^{k+1}=\rho {\left(A^{T}A\right)}^{-1}A^{T}\left(y-o^{k+1}-v^{k+1}-\frac{\tau^{k+1}}{\rho }\right).$$

### 4.2. SPOADMM Estimator

However, the $\ell_{p}$-norm residual term is nonconvex, and both the residual term and the regularization term are nonsmooth when 0≤p<1. Thus, its convergence is not guaranteed. To solve this problem, we propose to use a smoothing strategy to (9). Specifically, the $\ell_{1}$-norm regularization is smoothed as

(23) $${∥o∥}_{1,\varepsilon }=\sum_{i}{\left(o_{i}^{2}+\varepsilon^{2}\right)}^{\frac{1}{2}},$$

here, ε>0 is an approximate parameter,

(24) $$\lim_{\varepsilon \to 0}{∥o∥}_{1,\varepsilon }={∥o∥}_{1},$$

this means that with a smaller ε, ${∥o∥}_{1,\varepsilon }$ can exactly approximate the $\ell_{1}$-norm. With ε>0, the gradient of ${∥o∥}_{1,\varepsilon }$ is Lipschitz continuous. Therefore, the algorithm is guaranteed to converge if ρ is chosen sufficiently large (see Theorem 1). Using ${∥o∥}_{1,\varepsilon }$ as the regularization term, (9) becomes

(25) $$\begin{array}{c} \arg\min_{x,o}\frac{1}{2}{∥v∥}_{p}^{p}+\lambda {∥o∥}_{1,\varepsilon }\\ \;s.t.\;y-Ax-o=v. \end{array}$$

The corresponding augmented Lagrangian function is

(26) $$\begin{array}{c} L_{\varepsilon }\left(v,o,\tau ,x\right)=\frac{1}{2}{∥v∥}_{p}^{p}+{\lambda ∥o∥}_{1,\varepsilon }-<\tau ,y-Ax-o-v>+\frac{\rho }{2}{∥y-Ax-o-v∥}_{2}^{2}. \end{array}$$

The objective function of v-subproblem has not changed, only the range of *p* becomes 0<p<1. Correspondingly, the solution of v-subproblem becomes [45]

(27) $$v^{k+1}=\left\{\begin{array}{cc} \begin{array}{c} 0,\\ \left\{0,\mathrm{sign}\left(b^{k}\right)\beta \right\},\\ \mathrm{sign}\left(b^{k}\right)u^{k}, \end{array} & \left|\begin{array}{c} b^{k}\\ b^{k}\\ b^{k} \end{array}\right|\begin{array}{c} <\eta \\ =\eta \\ >\eta \end{array} \end{array}\right.,$$

where $\beta ={\left[\frac{1-p}{\rho }\right]}^{\frac{1}{2-p}}$, $\eta =\beta +\frac{p\beta^{p-1}}{2\rho }$, and $u^{k}$ is the solution of $h_{2}\left(u\right)=\frac{1}{2}pu^{p-1}+\rho u-\rho \left|b^{k}\right|=0$ over the region $\left(\beta ,\left|b^{k}\right|\right)$. The function $h_{2}\left(u\right)$ is convex, so, $u^{k}$ can be iteratively computed using Newton’s method when $\left|b^{k}\right|>\eta$. When p=0, $v^{k+1}$ can be solved by

(28) $$v^{k+1}=\left\{\begin{array}{cc} 0, & \left|b^{k}\right|<\sqrt{1/\rho }\\ b^{k}, & \;\mathrm{otherwise}\; \end{array}\right..$$

The o-subproblem becomes

(29) $$o^{k+1}=\arg\min_{o}\lambda {∥o∥}_{1,\varepsilon }+\frac{\rho }{2}{∥y-Ax^{k}-o-v^{k+1}-\frac{\tau^{k}}{\rho }∥}_{2}^{2}.$$

We can approximately solve this subproblem by linearizing the term ${∥o∥}_{1,\varepsilon }$. More precisely, at a given point $o^{k}$ we have

(30) $${∥o∥}_{1,\varepsilon }\approx {∥o^{k}∥}_{1,\varepsilon }+\langle o-o^{k},d\left(o^{k}\right)\rangle +\frac{L}{2}{∥o-o^{k}∥}_{2}^{2},$$

which results in the following closed-form solution by first-order optimality conditions

(31) $$o^{k+1}=\frac{\lambda Lo^{k}+\rho c^{k}-\lambda d\left(o^{k}\right)}{\lambda L+\rho },$$

where $d\left(o^{k}\right)=\nabla {∥o^{k}∥}_{1,\varepsilon }$ with $d{\left(o^{k}\right)}_{i}=o_{i}{\left(o_{i}^{2}+\varepsilon_{i}^{2}\right)}^{-\frac{1}{2}}$, L>0 is a proximal parameter.

**Theorem** **1.** *Suppose that ε>0, for any p>0 if*

(32) $$\rho >\frac{2L^{2}+2{\left(L+\frac{1}{\varepsilon }\right)}^{2}}{L-\frac{1}{2\varepsilon }}.$$

*Then, the sequence $\left\{v^{k},o^{k},\tau^{k},x^{k}\right\}$ generated by the SPOADMM converges to a stationary point of Formula (25).*

**Proof of Theorem 1.**  First, the Hessian of ${∥o∥}_{1,\varepsilon }$ is

(33) $$\nabla^{2}{∥o∥}_{1,\varepsilon }=\varepsilon^{2}\mathrm{diag}\left\{{\left(o_{1}^{2}+\varepsilon^{2}\right)}^{-\frac{3}{2}},\ldots ,{\left(o_{N}^{2}+\varepsilon^{2}\right)}^{-\frac{3}{2}}\right\}≺\frac{1}{\varepsilon }I_{m},$$

which implies that the gradient of ${∥o∥}_{1,\varepsilon }$ is $\frac{1}{\varepsilon }$-Lipschitz continuous, thus, we have

(34) $${∥o^{k+1}∥}_{1,\varepsilon }\leq {∥o^{k}∥}_{1,\varepsilon }+\langle o^{k+1}-o^{k},\nabla {∥o^{k}∥}_{1,\varepsilon }\rangle +\frac{1}{2\varepsilon }{∥o^{k+1}-o^{k}∥}_{2}^{2}.$$

Moreover, let $h\left(o\right)=\frac{\rho }{2}{∥y-Ax^{k}-o-v^{k+1}-\frac{\tau^{k}}{\rho }∥}_{2}^{2}$, the o-subproblem actually minimizes the following approximate object

(35) $$Q_{o^{k}}\left(o\right)=\langle o-o^{k},\nabla {∥o^{k}∥}_{1,\varepsilon }\rangle +\frac{L}{2}{∥o-o^{k}∥}_{2}^{2}+h\left(o\right).$$

Since $Q_{o^{k}}\left(o\right)$ is *L*-strongly convex, for any $o^{k}\in R^{n}$ we have

(36) $$Q_{o^{k}}\left(o^{k}\right)\geq Q_{o^{k}}\left(o^{k+1}\right)+\langle o^{k}-o^{k+1},\nabla Q_{o^{k}}\left(o^{k+1}\right)\rangle +\frac{L}{2}{∥\nabla^{2}Q_{o^{k}}\left(o^{k+1}\right)∥}_{2}^{2}.$$

From the definition of $o^{k+1}$ as a minimizer of $Q_{o^{k}}\left(o\right)$, we have $\nabla Q_{o^{k}}\left(o^{k+1}\right)=0$. Further, with $Q_{o^{k}}\left(o^{k}\right)=h\left(o^{k}\right)$, it follows from (35) and (36) that

(37) $$h\left(o^{k}\right)-L{∥o^{k+1}-o^{k}∥}_{2}^{2}\geq \langle o^{k+1}-o^{k},\nabla {∥o^{k}∥}_{1,\varepsilon }\rangle +h\left(o^{k+1}\right),$$

which together with (34) results in

(38) $$h\left(o^{k}\right)+{∥o^{k}∥}_{1,\varepsilon }-\left(L-\frac{1}{2\varepsilon }\right){∥o^{k+1}-o^{k}∥}_{2}^{2}\geq h\left(o^{k+1}\right)+{∥o^{k+1}∥}_{1,\varepsilon }.$$

Observe that the approximate o-subproblem actually minimizes $Q_{o^{k}}\left(o\right)$ in (36), whose minimum $o^{k+1}$ satisfies

(39) $$0=\nabla {∥o^{k}∥}_{1,\varepsilon }+L\left(o^{k+1}-o^{k}\right)-\rho \left(y-Ax^{k}-o^{k+1}-v^{k+1}-\frac{\tau^{k}}{\rho }\right).$$

Plugging (13) into (39) yields

(40) $$\nabla {∥o^{k}∥}_{1,\varepsilon }+L\left(o^{k+1}-o^{k}\right)=-\tau^{k+1}.$$

Then, it follows that

(41) $$\begin{array}{cc} {∥\tau^{k+1}-\tau^{k}∥}_{2}^{2} & \leq \left({∥\nabla {∥o^{k}∥}_{1,\varepsilon }-\nabla {∥o^{k-1}∥}_{1,\varepsilon }∥}_{2}+L{∥o^{k+1}-o^{k}∥}_{2}\right.{\left.+L{∥o^{k}-o^{k-1}∥}_{2}\right)}^{2}\\ \; & \leq {\left(\frac{1}{\varepsilon }{∥o^{k}-o^{k-1}∥}_{2}+L{∥o^{k+1}-o^{k}∥}_{2}+L{∥o^{k}-o^{k-1}∥}_{2}\right)}^{2}\\ \; & \leq 2L^{2}{∥o^{k+1}-o^{k}∥}_{2}^{2}+2{\left(L+\frac{1}{\varepsilon }\right)}^{2}{∥o^{k}-o^{k-1}∥}_{2}^{2}. \end{array}$$

From (38) and the definition of $L_{\varepsilon }$, we have

(42) $$L_{\varepsilon }\left(v^{k+1},o^{k+1},\tau^{k},x^{k}\right)-L_{\varepsilon }\left(v^{k+1},o^{k},\tau^{k},x^{k}\right)\leq -\left(L-\frac{1}{2\varepsilon }\right){∥o^{k+1}-o^{k}∥}_{2}^{2}.$$

It follows from (13), (41) and the definition of $L_{\varepsilon }$ that

(43) $$\begin{array}{cc} L_{\varepsilon }\left(v^{k+1},o^{k+1},\tau^{k+1},x^{k}\right) & -L_{\varepsilon }\left(v^{k+1},o^{k+1},\tau^{k},x^{k}\right)=\frac{1}{\rho }{∥\tau^{k+1}-\tau^{k}∥}_{2}^{2}\\ \; & \leq \frac{2L^{2}}{\rho }{∥o^{k+1}-o^{k}∥}_{2}^{2}+\frac{2{\left(L+\frac{1}{\varepsilon }\right)}^{2}}{\rho }{∥o^{k}-o^{k-1}∥}_{2}^{2}. \end{array}$$

Further, from the definition of $v^{k+1}$ and $x^{k+1}$ as a minimizer, respectively, we have

(44) $$L_{\varepsilon }\left(v^{k+1},o^{k},\tau^{k},x^{k}\right)-L_{\varepsilon }\left(v^{k},o^{k},\tau^{k},x^{k}\right)\leq 0,$$

(45) $$L_{\varepsilon }\left(v^{k+1},o^{k+1},\tau^{k+1},x^{k+1}\right)-L_{\varepsilon }\left(v^{k+1},o^{k+1},\tau^{k+1},x^{k}\right)\leq 0.\;$$

Summing (42)–(45), we can get that

(46) $$\begin{array}{cc} L_{\varepsilon }\left(v^{k+1},o^{k+1},\tau^{k+1},x^{k+1}\right) & -L_{\varepsilon }\left(v^{k},o^{k},\tau^{k},x^{k}\right)\\ \; & \leq \left(\frac{2L^{2}}{\rho }-L+\frac{1}{2\varepsilon }\right){∥o^{k+1}-o^{k}∥}_{2}^{2}+\frac{2{\left(L+\frac{1}{\varepsilon }\right)}^{2}}{\rho }{∥o^{k}-o^{k-1}∥}_{2}^{2}. \end{array}$$

Therefore, it can be concluded that if

(47) $$\rho >\frac{2L^{2}+2{\left(L+\frac{1}{\varepsilon }\right)}^{2}}{L-\frac{1}{2\varepsilon }},$$

which proved that the SPOADMM is convergent. □

## 5. Simulation Experiments

This section illustrates the effectiveness and robustness of the POADMM estimator and the SPOADMM estimator through simulations in the uniformly distributed noise by comparing them with the PLE estimator [18], the $\ell_{1}$-norm minimization estimator [46], the $\ell_{2}$-$\ell_{1}$ estimator [44], and the $\ell_{2}$-GMC estimator [32]. The $\ell_{1}$-norm minimization problem needs to be solved with CVX tools. Finally, the root-mean-squared-error (RMSE) is employed for performance comparison and is given by $\sqrt{\frac{1}{J}\overset{J}{\underset{j=1}{\Sigma }}{∥{\hat{x}}^{\left(j\right)}-T∥}^{2}}$, where ${\hat{x}}^{\left(j\right)}$ denotes the position estimated from the *j*-th Monte Carlo run.

**Figure 5:**
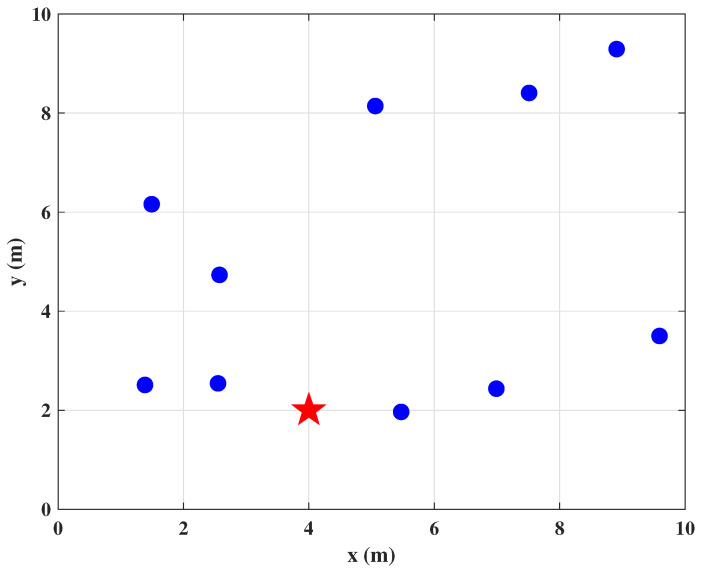
Sensor geometry.

Considering a simulated 2D AOA target localization geometry with 10 sensors randomly distributed in a 10 m × 10 m test area. The target sensor located at ${\left[4m,2m\right]}^{T}$. The geometric positions of sensors are shown in Figure 5, the blue dots represent sensors and the red star denotes the target. Each sensor is capable of measuring angles with respect to the target.

A total of J=500 Monte Carlo runs are carried out, and in each run which sensor measurements are outliers are assigned randomly. The maximum iteration number is set to 30. Since a good initialization in the nonconvex case is essential to obtain satisfactory performance for the SPOADMM estimator. It is recommended to use robust methods for initialization, and we use the POADMM with p=1. When p≥1, the POADMM is run with ρ=1 and the o-subproblem is updated via (20). When p≤1, the SPOADMM is run with $\rho =10^{3}$ and the o-subproblem is updated via (31) with $\varepsilon =10^{-3}$ and $L=\frac{1}{\varepsilon }$.

**Figure 6:**
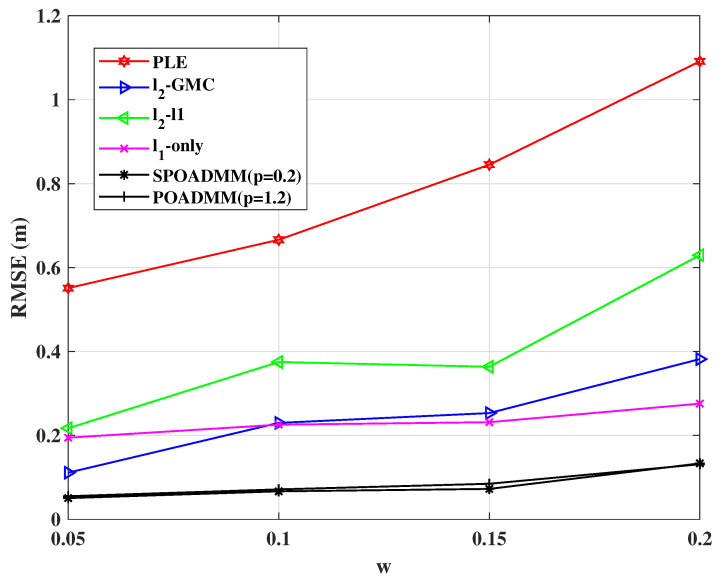
RMSE versus *w* in mixed noise for $\sigma^{2}=1$, λ=1.

Figure 6 shows the localization performance of the compared methods with different outlier probabilities *w* for $\sigma^{2}=1$ and λ=1. As shown in the figure, we can observe the PLE estimator, which without handling outliers, as expected, performs unreliably. Among the robust localization estimators, the $\ell_{2}$-$\ell_{1}$, the $\ell_{2}$-GMC, and the $\ell_{1}$-norm minimization exhibit worse performance than the proposed methods.

**Figure 7:**
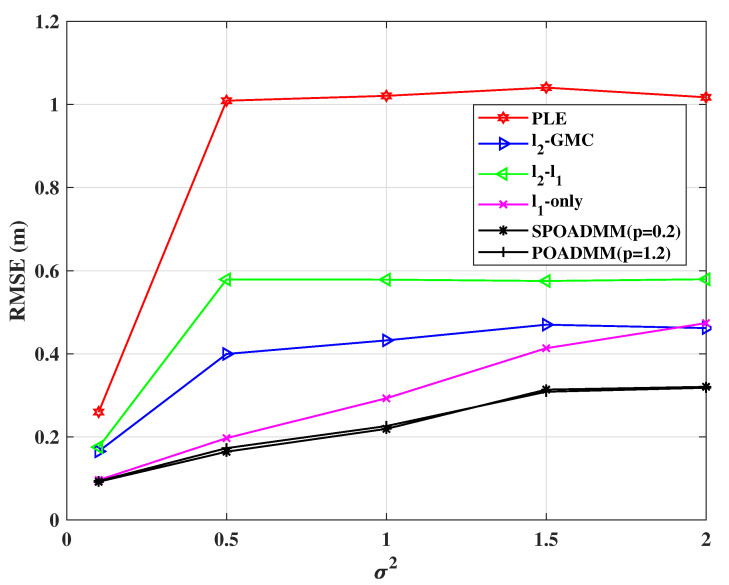
RMSE versus $\sigma^{2}$ in mixed noise for w=0.2, λ=1.

**Figure 8:**
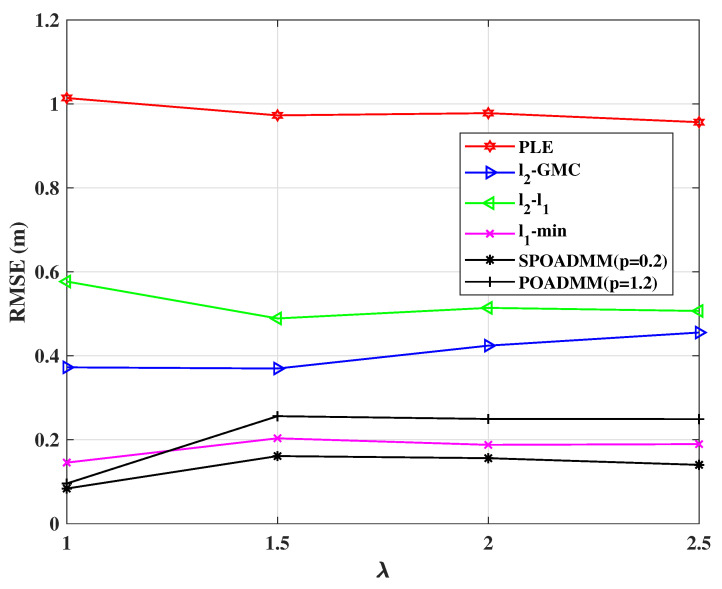
RMSE versus λ in mixed noise for w=0.2, $\sigma^{2}=0.5$.

Figure 7 and Figure 8 plot RMSEs performance of these estimators versus the variance of the measurement noise $\sigma^{2}$ and the regularization parameter λ, respectively. The results in the figures are obtained using the value of $\sigma^{2}$ and λ, respectively, which minimize RMSE. We increment $\sigma^{2}$ from 0.1 to 2 and λ from 1 to 2.5. The probability of outliers occurrence is w=0.2. It can be seen from Figure 7 that the localization performance of all methods decreases with the growth of $\sigma^{2}$, and the proposed POADMM and SPOADMM methods display lower RMSEs than others. In both figures, the $\ell_{1}$-norm minimization, the $\ell_{2}$-$\ell_{1}$, the $\ell_{2}$-GMC, the POADMM, and the SPOADMM distinctly outperform the PLE. This is due to the PLE being very sensitive to outliers. Although with the increase in λ in Figure 8, the POADMM exhibits poorer positioning performance. However, the RMSE of the SPOADMM is lower than others, which indicates the improvement of robust performance. It indicates that it is necessary to consider the case of 0≤p<1.

**Figure 9:**
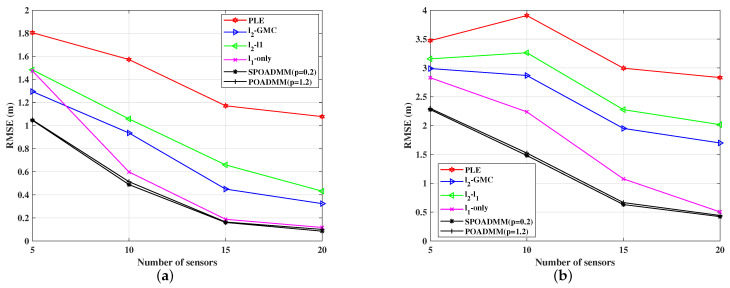
RMSE versus the number of sensors in mixed noise for w=0.2, $\sigma^{2}=1$, and λ=1. (**a**) w=0.2, $\sigma^{2}=1$, λ=1, and $T={\left[4,2\right]}^{T}$m. (**b**) w=0.2, $\sigma^{2}=1$, λ=1, and $T={\left[0,0\right]}^{T}$m.

Figure 9 compares RMSEs of these estimators versus the number of sensors *N* ranging from 5 to 20 for w=0.2, $\sigma^{2}=1$, and λ=1 when the target position is different. We observe that the localization performance of the compared methods can be improved as the number of sensors increases. Moreover, Figure 9 once again verifies the performance advantages of the POADMM and the SPOADMM methods compared to the PLE estimator, the $\ell_{2}$-$\ell_{1}$ estimator, the $\ell_{2}$-GMC estimator, and the $\ell_{1}$-norm minimization estimator in uniformly distributed noise for the target position at $T={\left[4,2\right]}^{T}$m. Note that, the proposed method always shows a slightly better localization performance with the number of sensors increasing when the target is located at ${\left[0,0\right]}^{T}$m. Its localization performance is slightly worse since it is farther from the test area than the target at ${\left[4,2\right]}^{T}$m.

## 6. Conclusions

This work introduced a robust algorithm for target localization, which employs the $\ell_{p}$-norm with 0≤p<2 as the measurement error and the $\ell_{1}$-norm as the regularization term. The algorithm can estimate outliers and target positions simultaneously in uniform distributed noise. Then, the POADMM estimator is developed to solve the optimization problem via incorporating the proximity operator into the framework of ADMM when 1≤p<2. To deal with the nonconvex and nonsmooth optimization problem when 0≤p<1, the SPOADMM estimator is finally proposed by applying a smooth strategy. Simulation results show that the proposed estimators have the capability to achieve distinctly better localization accuracy compared with existing robust algorithms. In the presence of highly impulsive measurement noise, the SPOADMM estimator (0≤p<1) is preferred. Along the lines of the current study, we will consider the problem of correlation between measurements under the influence of high impulse noise and evaluate the probability of outliers occurring at each AOA measurement in the future.

## Data Availability

Not applicable.

## Abbreviations

**Symbol Description**	
lowercase bold notation	column vectors
uppercase bold notation	matrices
$I_{m}$	an m×m identity matrix
〈·,·〉	inner product
${\left(\cdot \right)}^{T}$	matrix transpose operators
${\left(\cdot \right)}^{-1}$	matrix inverse operators
${∥x∥}_{p}={\left(\sum_{n}{\left|x_{n}\right|}^{p}\right)}^{1/p}$	the $\ell_{p}$-norm of x, $x\in R^{N}$
${∥x∥}_{1}=\sum_{n}\left|x_{n}\right|$	the $\ell_{1}$-norm of x, $x\in R^{N}$
${∥x∥}_{2}={\left(\sum_{n}{\left|x_{n}\right|}^{2}\right)}^{1/2}$	the $\ell_{2}$-norm of x, $x\in R^{N}$
∇f(·)	the gradient of the function f
$\nabla^{2}f\left(\cdot \right)$	the Hessian of the function f
*w*	the outlier occurrence probability
$\sigma^{2}$	the variance of the measurement noise
λ	the regularization parameter
